# Supplementary figures and images for: The distribution of insect pests and the associated loss of stored sorghum in the Kena district of Konso Zone, South-Western Ethiopia
Source: PLoS One. 2024 Jan 12;19(1):e0295833. doi: 10.1371/journal.pone.0295833 (PMC10786387; doi:10.1371/journal.pone.0295833)

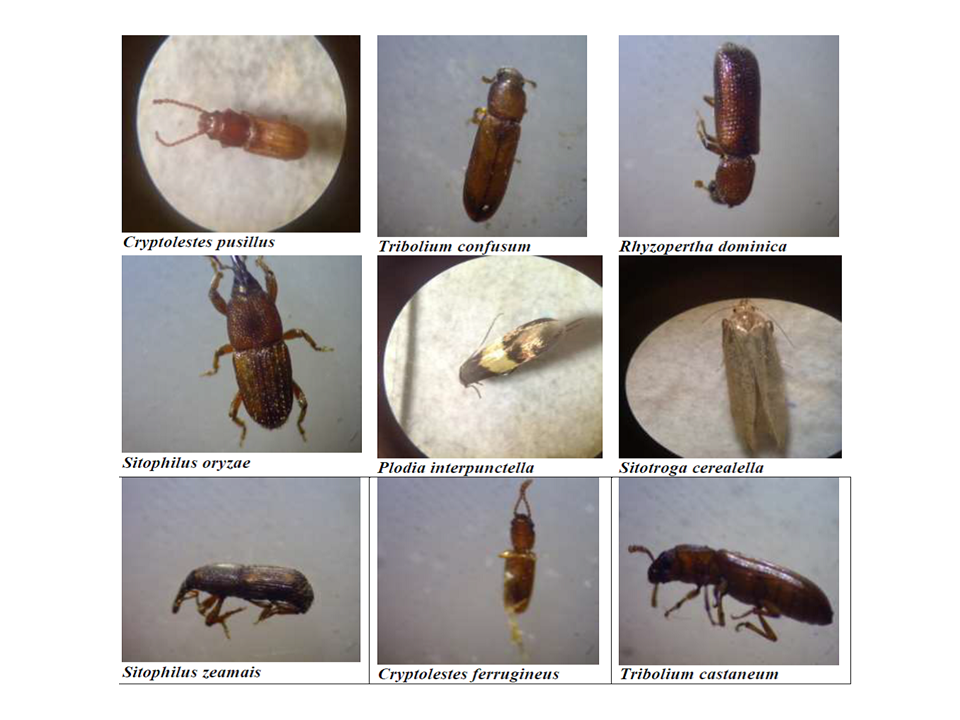

Supplement: S1 Fig — (TIF) [file pone.0295833.s001.tif]

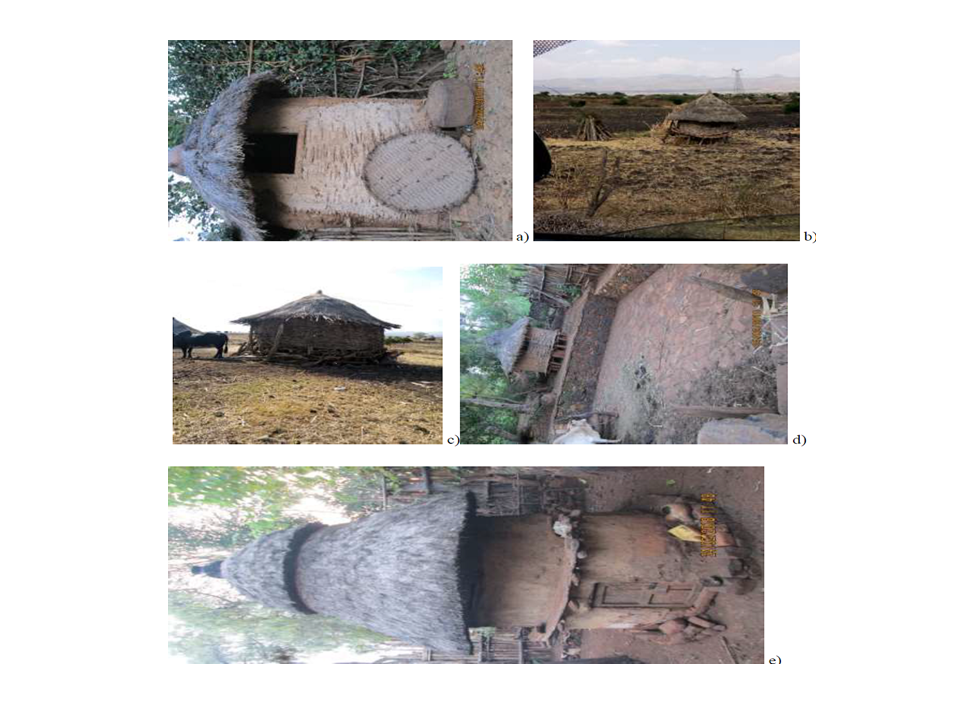

Supplement: S2 Fig — Partial views of pictures of barn sorghum storage structure (a-e). (TIF) [file pone.0295833.s002.tif]

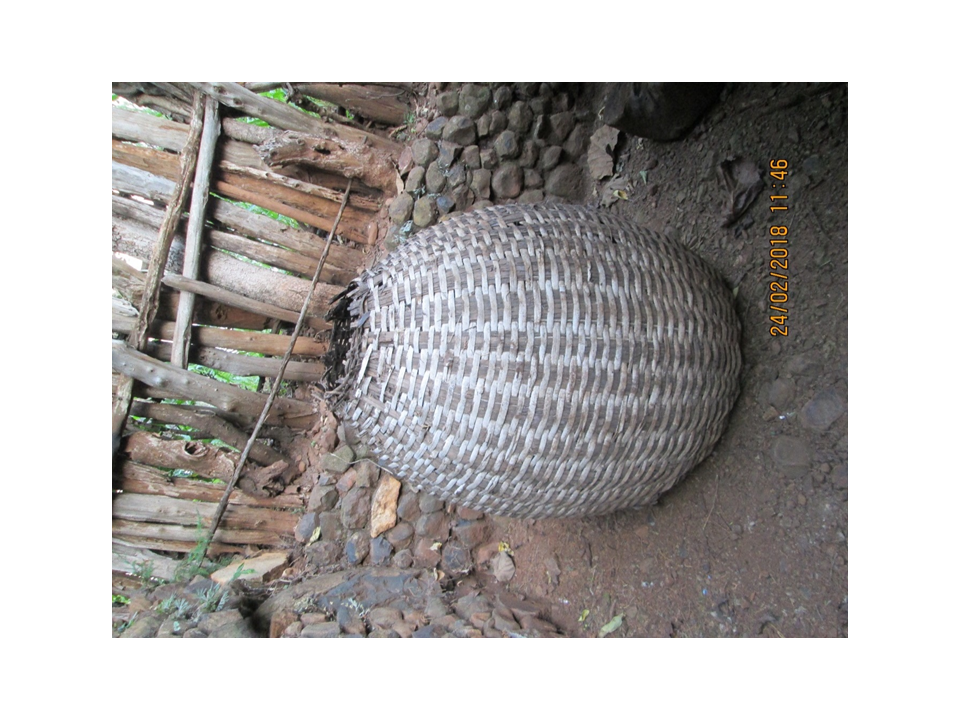

Supplement: S3 Fig — (TIF) [file pone.0295833.s003.tif]

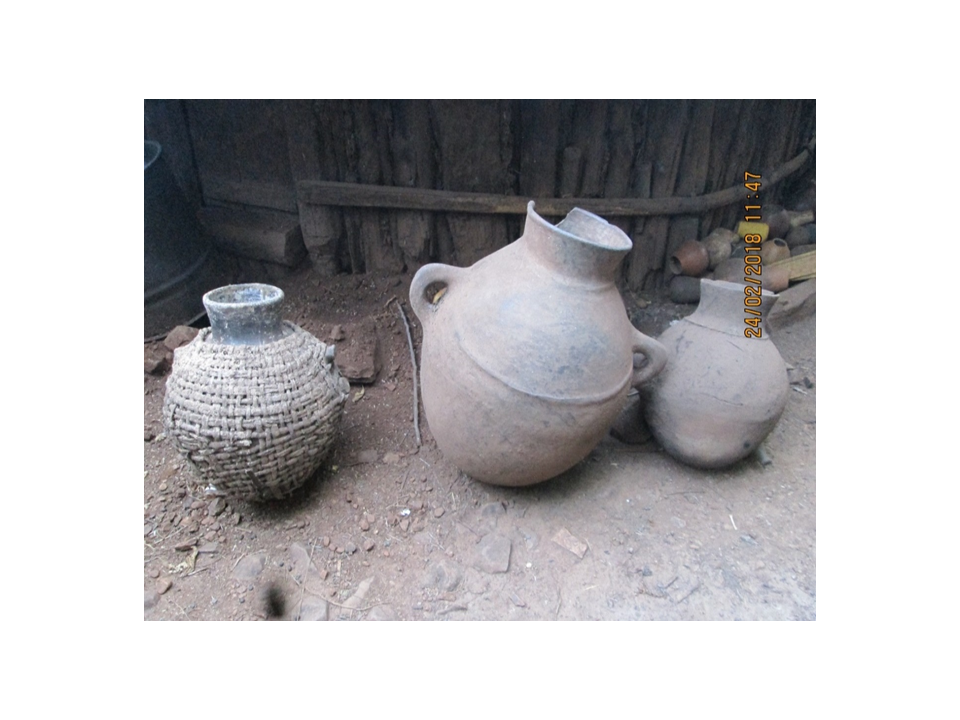

Supplement: S4 Fig — (TIF) [file pone.0295833.s004.tif]
