# Supplementary material for: The distribution of insect pests and the associated loss of stored sorghum in the Kena district of Konso Zone, South-Western Ethiopia
Source: PLoS One. 2024 Jan 12;19(1):e0295833. doi: 10.1371/journal.pone.0295833 (PMC10786387; doi:10.1371/journal.pone.0295833)
Supplement: S1 File — (PDF) [file pone.0295833.s005.pdf]

አርባ ምንጭ ዩኒቨርሲቲ
Arba Minch University
ተፈጥሮ ሳይንስ ኮሌጅ
College of Natural Scienses
ባዮሎጂ ት/ክፍል
DEPARTMENT OF BIOLOGY

ስልክ +251-046-881-1351

P.O.Box 21
ARBA MINCH, ETHIOPIA

Ref.No Bi.0.1/229/12
Date 27, Oct. 2018

To Sena District of Konso Zone

Fesha fown

This is to inform you that a student Ararso Gognsha thesis proposal that will be done in selected Kebeles of Kena District is aproved by apropirate proffecionals of the Depertement of Biology of Collage of Natural and Comptitional Sciences of Arba Minch University. Thus, the Depertement kindely ask to provide apropirate support that eneble him to collecte data on proposal entitled by Abundence and Management Practices of Insect Pests of Stored Sorghum (*Sorghum bicolour* L.) in Kena District of Konso Zone, Southern Ethiopia

አርባ ምንጭ ዩኒቨርሲቲ
Arba Minch University
የባዮሎጂ ት/ት ክፍል
Department of Biology
የተፈጥሮ ሳይንስ ኮሌጅ
College of the Natural Sciences

With best regards!

አለሙ ጩመዳ ኢፋ (ረዳት ፕሮፌሰር)
Alemu Chemeda Ifa (Asst. Professor)
የባዮሎጂ ት/ክፍል ኃላፊ
Head, Department of Biology
