# Supplementary material for: The distribution of insect pests and the associated loss of stored sorghum in the Kena district of Konso Zone, South-Western Ethiopia
Source: PLoS One. 2024 Jan 12;19(1):e0295833. doi: 10.1371/journal.pone.0295833 (PMC10786387; doi:10.1371/journal.pone.0295833)
Supplement: Attachment — Submitted filename: Comments to The Distribution of Insect Pests, the associated Loss of Stored Sorghum and Food Insecurity in the Kena District of Konso Zone, South Western Ethiopia.pdf [file pone.0295833.s006.pdf]

Comments to: The Distribution of Insect Pests, the associated Loss of Stored Sorghum and Food Insecurity in the Kena District of Konso Zone, South Western Ethiopia.

General Comments

1. This is a good paper that adds to the literature. However, it does need a moderate to a significant amount of revision. There is a mismatch between the abstract/introduction, the beginning of the discussion, and the Conclusion.

2. The paper approach different topics. It has sections on the presence of different plagues, then grain damage and type of storage. But the topics are not well connected in the text. It would be good if the authors connect these different themes using a causal/effect to explain the relation.

3. The number of farmers (sampling) is not consistent across the text.

4. Some rewriting and clarification are required. I provide input in the specific comments.

5. It is important to add some definitions to the first part of the paper to clarify the context—for example, bamboo bin storage and barn storage, among others. (I will put more details in the specific comments).

6. When the authors mention “inappropriate storage facilities” it refers to the “a good condition” of this type of storage? (because if it’s the actual condition, this “inappropriate storage facility” is going to fail anyway), or it refers to the current status of these facilities?

7. Discussion. The lead paragraph/section of the discussion should provide analysis or summary of key Results. That will make the subsequent paragraph merger into the paper better - currently, the focus of the discussion is an abrupt change from the previous sections.

8. The paper doesn’t do an analysis on food insecurity, so I will suggest removing that from the title of the paper.

Specific comments:

Line 19: Are the losses in quantity, quality, or both?

Line 81: is this one harvest storage period? or many harvest periods?

Line 87: farmers stores refer to farmer storage or an intermediary? This term is not clear.

Line 91: The total sample was 1 kg? or was 1kg from each farmer store?

Line 95: it is not clear how many samples were taken. So, the authors took samples from all the farmers each month? or the samples from farmers were taken over a nine-month period? If is the second, what criteria was used to select who was the first? And how is this not affecting the presence of plagues?

Line 98 -104: this paragraph is the same as the previous one

Line 108-109: Does the 100 grams refer to grain sample or insects' sample?

Line 131-132: in previous paragraphs, authors talked about 360 representative farmers storage. This sentence talks about 108 bamboo bins and 108 barns.

Line 136- 144: This sample is a different sample from the 1 kg sample?

Line 136- 144: In the calculation of the percentage of grain damage, are the authors only included the grain damage by insects? Are they excluded grain damage by fungus or humidity? If this is the case, the calculation of grain losses need to specify that is not the total grain losses.

Line 195-196: It should be good to add a description of the climatic characteristics of the study sites to understand the differences in the presence of plagues.

Line 234-235: It would be good to add a description of these different types of storage. A description is added in the discussion (lines 330-333). Although this description is unclear, it would be good to move it to the introduction.
