# Supplementary material for: The distribution of insect pests and the associated loss of stored sorghum in the Kena district of Konso Zone, South-Western Ethiopia
Source: PLoS One. 2024 Jan 12;19(1):e0295833. doi: 10.1371/journal.pone.0295833 (PMC10786387; doi:10.1371/journal.pone.0295833)
Supplement: Attachment — Submitted filename: Response to Reviewers..docx [file pone.0295833.s007.docx]

**Revision made on reviewers’ comments**

First I would like to thank all the reviewers for their critical, constructive, and valuable comments that enrich the manuscript to the scientific standard. I would like also to thank the editors, including the editor in chief for their kind corrections, supports, and assistance. Below, I tried to indicate revisions that I made for reviewers’ and editor comments with different color.

**General reflection or revision made on comments rose**

- Green color highlighted revisions in the different parts of thee manuscripts are addressed are addressed for Reviewer 1 comments
- All red color highlighted revisions in the text, tables, and figures among others are addressed for Reviewer 2 comments
- Blue color highlighted revisions in the manuscript are addressed for reviewer 3 comments
- Yellow color highlighted revisions in the manuscript are addressed for editor comments; removing figure 1 & re arranging the sequence of the rest of figures accordingly
- Besides, I request editor to exclude S table 1 & 2, as these are included with in the text based reviewer 2 comment

Generally, I tried my best to address the comments raised by reviewers and editor in the whole document or manuscript.

**Specific reflection or revision made on comments raised**

| sn | Commenter | Comments raised by editor/reviewers | Revision addressed | Remark |
| --- | --- | --- | --- | --- |
| 1 | Editor | You may seek permission from the original copyright holder of Figure or remove it | Removed, the rest of the figures numbers are rearranged | If I am confused in understanding the comment, I am ready to accept any comments that will be raised |
|  |  | Please ensure that your manuscript meets PLOS ONE's style requirements | Tried to check it & ensured | I will accept any comments that will be raised regarding this, if it is not sufficiently addressed |
|  |  | If you wish to make changes to your Data Availability  statement, please describe these changes in your cover letter and we will update your Data Availability  statement to reflect the information you provide | Not changed | If the already existing one is not correct I will provide any time up on the editor request |
| 3 | Reviewer #1: | The paper doesn‘t do an analysis on food insecurity, so I will suggest removing that from the title of the paper. | removed |  |
|  |  | There is a mismatch between the abstract/introduction, the beginning of the discussion, and the Conclusion. | I tried to address it as highlighted with green color in its abstract/introduction, the beginning of the discussion, and the Conclusion. | I will accept any comments that will be raised regarding this, if it is not sufficiently addressed |
|  |  | The different topics are not well connected in the text. It would be good if the authors connect these different themes using a causal/effect to explain the relation. | I tried to address it as highlighted with green color in its | I will accept any comments that will be raised regarding this, if it is not sufficiently addressed |
|  |  | The number of farmers (sampling) is not consistent across the text | I tried to address it as highlighted with green color in its | I will accept any comments that will be raised regarding this, if it is not sufficiently addressed |
|  |  | It is important to add some definitions to the first part of the paper to clarify the context—for example, bamboo bin storage and barn storage, among others. (I will put more details in the specific comments). | addressed as highlighted with green color in its |  |
|  |  | When the authors mention “inappropriate storage facilities” it refers to the “a good condition” of this type of storage? | I tried to address it as highlighted with green color by replacing it with better term |  |
|  |  | Discussion. The lead paragraph/section of the discussion should provide analysis or summary of key Results. That will make the subsequent paragraph merger into the paper better | tried to address it as highlighted with green color | I will accept any comments that will be raised regarding this, if it is not sufficiently addressed |
|  |  | Are the losses in quantity, quality, or both? | I tried to address it as highlighted with green color by explain it as quantitative |  |
|  |  | this one harvest storage period? or many harvest periods | I tried to address it as highlighted with green color |  |
|  |  | farmers stores refer to farmer storage or an intermediary? This term is not clear. | I tried to address it as highlighted with green color | I will accept any comments that will be raised regarding this, if it is not sufficiently addressed |
|  |  | The total sample was 1 kg? or was 1kg from each farmer store? | I tried to address it as highlighted with green color | I will accept any comments that will be raised regarding this, if it is not sufficiently addressed |
|  |  | So, the authors took samples from all the farmers each month? or the samples from farmers were taken over a nine-month period? | I tried to address it as highlighted with green color | I will accept any comments that will be raised regarding this, if it is not sufficiently addressed |
|  |  | Line 98 -104: this paragraph is the same as the previous one | tried to address it as highlighted with green color | I will accept any comments that will be raised regarding this, if it is not sufficiently addressed |
|  |  | Does the 100 grams refer to grain sample or insects’ sample | tried to address it highlighted with green color |  |
|  |  | in previous paragraphs, authors talked about 360 representative farmers storage. This sentence talks about 108 bamboo bins and 108 barns. | tried to address it as highlighted with green color through clarification | I will accept any comments that will be raised regarding this, if it is not sufficiently addressed |
|  |  | In the calculation of the percentage of grain damage, are the authors only included the grain damage by insects | tried to address it as highlighted with green color through clarification | I will accept any comments that will be raised regarding this, if it is not sufficiently addressed |
|  |  | It should be good to add a description of the climatic characteristics of the study sites to understand the differences in the presence of plagues. | Addressed as highlighted with green color |  |
|  |  | It would be good to add a description of these different types of storage under introduction | Addressed as highlighted with red color | I will accept any comments that will be raised regarding this, if it is not sufficiently addressed |
| 3 | Reviewer #2: | It is very unclear what has  already been done, what gap in the literature the paper would fill, and how it can contribute to developing  more effective interventions. The authors should expand on the introduction or add an additional ‗literature review‘/ background section before the ‗material and methods‘ section. | Addressed as highlighted with red color | I will accept any comments that will be raised regarding this, if it is not sufficiently addressed |
|  |  | The research was carried out over 2 years, but at the same time it is said that this is the ‗sorghum storage  period‘. So, I guess Sorghum is stored the year round? Why then mentioning that this is the specific  ‗storage period‘? on the same page, you mention that samples were collected over a 9-month period (also | Addressed as highlighted with red color | I will accept any comments that will be raised regarding this, if it is not sufficiently addressed |
|  | Reviewer #2: | Clarify concepts of ‗agricultural district and kebele staff | Addressed as highlighted with red color |  |
|  |  | Better clarify the use of some terms or avoid using them : what is ‗each sampling date? what is the  ‗essential information‘? what do you mean by ‗complete mixing‘: | Addressed as highlighted with red color |  |
|  |  | Table 1: what do you mean by ‗distribution‘? ‗mean weight‘ of grains affected by insect pests? | Addressed as highlighted with red color | I will accept any comments that will be raised regarding this, if it is not sufficiently addressed |
|  |  | You jump back and forth from summary statistics to regression frameworks (ex. Table 6 are again  summary statistics after regressions in tab 4). Start with summary statistics, and then move on to  regressions. | Addressed as highlighted with red color as far as I understand the comment | If I am confused in understanding the comment, I am ready to accept any comments that will be raised |
|  |  | What is the added value of running several simple linear regressions? It is obvious that climate influences  pests (I assume that this has been proven by the literature) so it is rather more interesting to see how  different types of storage influence pests, by controlling for climate/ kebele fixed effects. So rather, run a multiple linear regression model and control for kebeles, climate and storage type | tried to address it as highlighted with red color | If I am confused in understanding the comment, I am ready to accept any comments that will be raised |
|  |  |  | If regression are not necessary the reviewer & editor can exclude it |  |
| 3 | Reviewer #2: | Figure 3 and tables S1, S2 are not easy to find: consider placing them in the main document if you describe them in the text. Lines 248: what do you mean by ―1=Bamboo bin storage‖ and 1.8= Barn storage? | tried to address it as highlighted with red color | If I am confused in understanding the comment, I am ready to accept any comments that will be raised |
|  |  | were significant in both the commonly used farmer‘s traditional storage‘… what are you  comparing here? Unclear.  o Significance level notations via ‗lower case letters‘ in the tables is very uncommon and difficult to follow. Stick to the **? | tried to address it as highlighted with red color | If I am confused in understanding the comment, I am ready to accept any comments that will be raised |
|  |  | variable ‗storage type‘  what type of storage are you looking at ? If this is a categorical variable,  you have to transform it into dummies.. or clarify what you are comparing if this is a dummy already.  Statements in line 277 are difficult to interpret without the full picture on table 7 | tried to address it as highlighted with red color | If I am confused in understanding the comment, I am ready to accept any comments that will be raised |
|  |  | To make this paper more interesting (or even find an added value of this paper), it would be good to explore interactions between climate conditions and storage methods: how do different storage methods perform in different agro-climatic contexts/ changing agro-environmental conditions due to climate change?  Try to explore heterogeneity by kebele? | tried to address it as highlighted with red color & edited with track change  It is for this comment that the last table (Table 9.) and the associated result and figures are added or included in the manuscript | If I am confused in understanding the comment, I am ready to accept any comments that will be raised |
|  |  | what is your contribution? What do you add to this existing evidence?  o Lines 343/344: can you conclude this? Are the storage methods that you assess and that perform better associated with increased ventilation?  o Line 352: your results do not really allow you to say anything about the ‗planning management strategies‘.  The same holds true for ‗awareness creation‘ in line 389  Other comments:  - Structure of the article: the use of different size/ formats of the titles and subtitles would facilitate the  reading (eg. It is now unclear whether ‗the study design and sampling procedure‘ is a subtitle of ‗Materials  and Methods‘ or at the same level)  - Consider revising the English of the manuscript | tried to address it as highlighted with red color, especially at the back ground part, in the meantime of addressing reviewer 1 comment  corrected  removed  corrected  corrected | If I am confused in understanding the comment, I am ready to accept any comments that will be raised |
| 4 | Reviewer #3 | Some more discussion on implications of the findings for the rest of the country and Africa would be useful.  They indicate that losses are enormous in the  introduction (50% - there is little solid evidence for these findings) while they themselves find only 12%.  The authors could refer to the global debate on the size of these post-harvest losses  The text is confusing regarding the period that is studied exact  understand that quite some farmers in Ethiopia use fumigants and other chemical means | tried to address all as highlighted with blue color | If I am confused in understanding the comment, I am ready to accept any comments that will be raised |
|  |  | Losses can be avoided but there are often costs involved to avoid those, such as improved storage, chemicals, and labor. It would be good if the authors would discuss more costs and benefits | tried to address all as highlighted with blue color | If I am confused in understanding the comment, I am ready to accept any comments that will be raised |
|  |  | The text is sloppy in a number of places and it would be good for the authors to carefully read through thetext before it is finalized. | tried to address all as highlighted with blue color | If I am confused in understanding the comment, I am ready to accept any comments that will be raised |

Best regards!
