# Supplementary material for: The distribution of insect pests and the associated loss of stored sorghum in the Kena district of Konso Zone, South-Western Ethiopia
Source: PLoS One. 2024 Jan 12;19(1):e0295833. doi: 10.1371/journal.pone.0295833 (PMC10786387; doi:10.1371/journal.pone.0295833)
Supplement: Attachment — Submitted filename: Review PONE 2.docx [file pone.0295833.s008.docx]

**Comments to the authors:**

I re-confirm that the paper addresses an important topic that is at the heart of the public policies targeting poverty reduction and tackling food insecurity. The data definitely have some potential. Yet, I still have concerns about the analysis that is carried out and the contribution of this paper.

Comments:

- The fit in the literature has been improved. Still, I think the authors could be clearer from the very beginning on what they do and what their added value is (just as an example, the authors describe the type of storage methods in the discussion – from line 433. This is the core of the paper and should be clear from the very beginning)
- Analysis & results:
  - I am puzzled by the way some analyses are carried out and thus the interpretation of the results:
    - kebele seems to be included as a continuous variable (e.g. Table 4) but these are dummies and should be included as such (interpretation is otherwise impossible). Same for ‘storage type’ what is compared against what? This also does not become clear in the description of the results
  - Regression and output tables are still reported in an unconventional way, but maybe this is an issue of a different disciplines. Yet, to get a picture of individual effects of climate versus storage, the analyses should be done jointly. Ideally by also adding interaction effects. Also, how do the authors disentangle between agroclimatic effects and simple kebele effects? Did they combine different kebeles with similar agro-climates?
  - Why not including duration of storage or storage management practices in the analysis (mentioned in line 427)? This could have really added some value..
- Discussion:
  - A lot of repetitions. As far as I understand there is two main messages:
    - Lower losses in barn storages (versus bamboo)
    - Lower losses in highlands

This should be stressed and repetitions avoided.
