# Supplementary material for: The distribution of insect pests and the associated loss of stored sorghum in the Kena district of Konso Zone, South-Western Ethiopia
Source: PLoS One. 2024 Jan 12;19(1):e0295833. doi: 10.1371/journal.pone.0295833 (PMC10786387; doi:10.1371/journal.pone.0295833)
Supplement: Attachment — Submitted filename: Response to Reviewers..docx [file pone.0295833.s009.docx]

**Revision made on reviewers’ comments**

First I would like to thank all the reviewers for their critical, constructive, and valuable comments that enrich the manuscript to the scientific standard. I would like also to thank the editors, including the editor in chief for their kind corrections, supports, and assistance. Below, I tried to indicate revisions that I made for reviewers’ and editor comments with different color.

**General reflection or revision made on comments rose**

- all Green, red and yellow color highlighted parts in the document are the revised parts based on reviwer 2 comments and for the gramer and language problems inprovement

Generally, I tried my best to address the comments raised by reviewer 2.

Comments by reviewer 2

Comment 1. I think the authors could be clearer from the very beginning on what they do and what their added value is (just as an

example, the authors describe the type of storage methods in the discussion – from line433. This is the core of the paper and should be clear from the very beginning)

- Response-I tried to address it as indicated in the introduction and methodology

Comment 2. I am puzzled by the way some analyses are carried out and thus the interpretation of the results:

 kebele seems to be included as a continuous variable (e.g. Table 4) but these are dummies and should be included as such (interpretation is otherwise impossible).

- Response- tis part is also tried to improved

Comment 3. Same for ‘storage type’ what is compared against what? This also does not become clear in the description of the results

- Response- tis part is also tried to improved

Comment 4. Regression and output tables are still reported in an unconventional way, but maybe this is an issue of different disciplines. Yet, to get a picture of individual effects of climate versus storage, the analyses should be done jointly.

- Response- it is done jointly

Comment 4. Also, how do the authors disentangle between agroclimatic effects and simple kebele effects? Did they combine different

kebeles with similar agro-climates?

- Response- yes

Comment 5. Why not including duration of storage or storage management practices in the

analysis (mentioned in line 427)? This could have really added some value

- Response- Management practices are included

Comment 5. Discussion:

o A lot of repetitions. As far as I understand there is two main messages:

 Lower losses in barn storages (versus bamboo)

 Lower losses in highlands

This should be stressed and repetitions avoided.

- Response- This also tried to addressed

The reviser or editor can avoid those parts they consider irrelevant or repeated in cases if my response is not satisfactory

Best regards!
